# Long noncoding RNA CRART16 confers 5-FU resistance in colorectal cancer cells by sponging miR-193b-5p

**DOI:** 10.1186/s12935-021-02353-5

**Published:** 2021-11-29

**Authors:** Jingui Wang, Xiaoqian Zhang, Junling Zhang, Shangwen Chen, Jing Zhu, Xin Wang

**Affiliations:** 1 Department of General Surgery, Peking University First Hospital, NO. 8 Xishiku Street, Xicheng, Beijing, 100034 People’s Republic of China; 2 Department of Colorectal Surgery, National Cancer Center/Cancer Hospital, Chinese Academy of Medical Sciences, No. 17, Panjiayuan Nanli, Chaoyang, Beijing, 100021 People’s Republic of China

**Keywords:** Long noncoding RNA, CRART16, 5-FU, Resistance, miR-193b-5p, HMGA2, MAPK

## Abstract

**Background:**

The emergence of chemoresistance to 5-fluorouracil (5-FU)-based chemotherapy is the main cause of treatment failure in advanced and metastatic colorectal cancer (CRC) patients. Long noncoding RNAs (lncRNAs) have been reported to be involved in 5-FU resistance. Previously, we first detected that lncRNA cetuximab resistance-associated RNA transcript 16 (CRART16) could contribute to cetuximab resistance by upregulating V-Erb-B2 erythroblastic leukemia viral oncogene homologue 3 (ERBB3) expression by sponging miR-371a-5p in CRC cells. The current study aimed to explore the role of CRART16 in acquired 5-FU resistance in CRC cells and its possible mechanism.

**Methods:**

Quantitative real-time PCR (RT-qPCR) was used to measure the expression levels of CRART16 in a 5-FU-resistant CRC cell subline (SW620/5-FU) and the parent cell line. Lentivirus transduction was performed to establish SW620 and Caco-2 cells stably overexpressing CRART16. Cell Counting Kit-8 (CCK-8) assays and colony formation assays were applied to measure cell chemosensitivity to 5-FU. Flow cytometric and immunofluorescence staining were adopted to assess cell apoptosis induced by 5-FU. The dual-luciferase reporter assay was used to validate the direct interactions between CRART16 and miR-193b-5p and between miR-193b-5p and high-mobility group AT-hook-2 (HMGA2). The expression levels of HMGA2, apoptosis-associated proteins and p-ERK were examined by western blotting. The statistical differences within any two groups were used Student’s t test.

**Results:**

CRART16 was upregulated in SW620/5-FU cells. Overexpression of CRART16 reduced the sensitivity of CRC cells to 5-FU by attenuating apoptosis. In addition, CRART16 promoted 5-FU resistance by suppressing the expression of miR-193b-5p. Furthermore, CRART16 modulated the expression of HMGA2 by inhibiting miR-193b-5p and activated the MAPK signaling pathway.

**Conclusions:**

CRART16 confers 5-FU resistance in CRC cells through the CRART16/miR-193b-5p/HMGA2/MAPK pathway.

## Background

Colorectal cancer (CRC) is among the most common cancers worldwide, ranking third in terms of incidence but second in terms of mortality [1]. After decades of research and development, comprehensive treatment based on surgical resection has become the main treatment mode of CRC; the involved approaches include endoscopic and surgical local excision, adjuvant chemotherapy, downstaging preoperative chemoradiotherapy, extensive surgery for locoregional and metastatic disease, local ablative therapies for metastases, palliative surgery and chemotherapy, targeted therapy, and immunotherapy. Among them, drug therapy has developed the most rapidly, significantly improving the prognosis of advanced and metastatic colorectal cancer (mCRC) patients [2]. Despite the fact that research has entered into the era of targeted therapy and immunotherapy, 5-fluorouracil (5-FU) is still the first-line drug and plays an irreplaceable role in the treatment of advanced CRC and mCRC [3].

5-FU, an analogue of uracil that was first synthesized by Heidelberger et al. [4] in 1957, eventually inhibits the synthesis of purines by inhibiting thymidylate synthase (TS) activity in vivo, resulting in the inhibition of DNA replication and repair. In addition, the incorporation of 5-FU-derived nucleosides into RNA fractions interferes with RNA synthesis and function [5]. However, it has been documented that a considerable percentage of CRC patients and nearly half of mCRC patients experience disease progression during the course of 5-FU-based chemotherapy [6, 7]. Both primary and acquired chemoresistance are responsible for treatment failure and limit the clinical application of 5-FU [8]. Although many efforts have been made to reveal the molecular mechanisms underlying chemoresistance to 5-FU in CRC, novel therapeutic targets have yet to be identified.

As new potential regulators in various cellular processes, long noncoding RNAs (lncRNAs), noncoding RNAs (ncRNAs) withs length longer than 200 nucleotides and without protein-coding capacity, have recently attracted growing interest in different cancer types [9]. Several studies have indicated that some lncRNAs play an important role in drug resistance [10–13]. LncRNA UCA1 has been reported to contribute to cisplatin/gemcitabine resistance in vitro and vivo via CREB, which modulates miR-196b-5p in bladder cancer [14]. Similarly, lncRNA XLOC_006753 has been proven to promote multidrug resistance in vitro via the PI3K/AKT/mTOR signaling pathway in gastric cancer [15]. To date, few studies have focused on lncRNAs in CRC 5-FU chemoresistance [16]. Previously, we first found that lncRNA cetuximab resistance-associated RNA transcript 16 (CRART16) promotes cetuximab resistance by enhancing V-Erb-B2 erythroblastic leukemia viral oncogene homologue 3 (ERBB3) expression through miR-371a-5p in CRC cells [17]. However, the role of lncRNA CRART16 in CRC cell 5-FU resistance needs to be further investigated.

In this study, we found that the expression level of CRART16 was upregulated in SW620/5-FU cells. Further mechanistic investigation demonstrated that when it was overexpressed, CRART16 enhanced the 5-FU resistance of CRC cells and upregulated high-mobility group AT-hook-2 (HMGA2) expression by sponging miR-193b-5p. Moreover, we revealed that the MAPK signaling pathway was activated by CRART16.

## Methods

### Cell lines

HEK-293 T and human CRC lines (SW620, Caco-2) were purchased from the Cancer Institute of the Chinese Academy of Medical Science and cultured in Dulbecco’s modified Eagle’s medium (DMEM, Thermo Fisher Scientific, MA, USA) supplemented with 10% fetal bovine serum (FBS, Thermo Fisher Scientific, MA, USA) and 1% penicillin/streptomycin (Thermo Fisher Scientific, MA, USA). The 5-FU-resistant CRC subline (SW620/5-FU) was developed by exposing parental cells to 5-FU in stepwise increasing concentrations for approximately 6 months. SW620/5-FU cells were maintained in DMEM supplemented with 10 μM 5-FU. All cells were grown at 37 °C in an incubator with 5% CO2.

### RNA extraction and quantitative real-time PCR (RT-qPCR) analyses

Total RNA was isolated from cultured cells using TRIzol Reagent (Invitrogen, Carlsbad, CA, USA) following the manufacturer’s instructions. For miRNA quantification, reverse-transcribed complementary DNA was synthesized from 2 µg extracted total RNA using TransScript miRNA RT Enzyme Mix (Transgen Biotech, Beijing, China) and amplified with TransStart TIP Green qPCR SuperMix (Transgen Biotech, Beijing, China) with normalization to U6. For lncRNA and mRNA detection, RNA was reverse-transcribed with random primers using the RevertAid First Strand cDNA Synthesis Kit (Thermo Fisher Scientific, MA, USA) and amplified with PowerUp™ SYBR™ Green Master Mix (Thermo Fisher Scientific, MA, USA) with glyceraldehyde 3-phosphate dehydrogenase (GAPDH) as an internal control. RT-qPCR was performed with a 7500 Real-Time PCR System (Applied Biosystems, Germany). The relative RNA expression levels were calculated with the 2^−∆∆Ct^ method normalized by internal control. The RT-qPCR experiment in this study followed the MIQE guidelines [18]. The primer sequences used in the study are listed in Table 1.

**Table 1:** Primer sequences used for RT-qPCR

Gene	Sequence of the primers
lncRNA CRART16, forward primer	5′-TGATAGTGAGGCCTCCTGCAA-3′
lncRNA CRART16, reverse primer	5′-CTGGAGTTCTGCAGGTTCCTTT-3′
miR-193b-5p, forward primer	5′-CGGGGTTTTGAGGGCGAGATGA-3′
U6, forward primer	5′-GCAAGGATGACACGCAAATTC-3′
HMGA2, forward primer	5′-GCAGCAAAAACAAGAGTCCCTCTA-3′
HMGA2, reverse primer	5′-GCCTCTTGGCCGTTTTTCTC-3′
GAPDH, forward primer	5'-GCACCGTCAAGGCTGAGAAC-3'
GAPDH, reverse primer	5'-ATGGTGGTGAAGACGCCAGT-3'

### Lentivirus transduction

The plasmid pCDH-CMV-MCS-EF1-GFP + Puro containing full-length CRART16 cDNA (Mailgene, Beijing, China) was constructed to stably overexpress the expression of CRART16 in SW620 and Caco-2 cells by lentivirus transduction as we previously described [17]. The cells were named SW620-CRART16 and Caco-2-CRART16, and their negative control cells were called SW620-NC and Caco-2-NC, respectively.

### Dual luciferase reporter gene assay

The full-length CRART16 and HMGA2 3′ untranslated regions (3′-UTRs) were inserted into the pmiR-RB-Report™ vector to construct luciferase reporter vectors (RiboBio, Guangzhou, China). HEK-293 T cells were seeded in 96-well plates and cultured overnight. HEK-293 T cells were co-transfected with 0.1 μg WT vector or empty vector and 50 nM miR-193b-5p mimics (RiboBio, Guangzhou, China) or negative controls using Lipofectamine 3000 (Invitrogen, Carlsbad, CA, USA) according to the manufacturer’s protocols. Forty-eight hours after co-transfection, the Dual‐Luciferase Reporter Assay System (Cat. E2920, Promega, Madison, Wisconsin, USA) was used to measure the luciferase activity, and all experiments were carried out in triplicate.

### Cell viability assay

For dose–effect curve depiction, the cells were seeded into 96-well plates and cultured overnight, and then the culture medium was replaced with fresh culture medium containing different concentrations of 5-FU. After 48 h of incubation, Cell Counting Kit-8 (CCK-8; Bimake, Shanghai, China) assays were performed to detect cell viability. The optical density at 450 nm was measured using a spectrophotometer at different time points. Each experiment was performed thrice.

### Colony formation assay

Transfected cells were seeded (300 cells per well) into 6-well plates and cultured overnight. Then, the culture medium was replaced with fresh culture medium containing 5-FU. The medium was changed every 5 days. After 10–14 days, colonies could be clearly observed. The colonies were fixed with methanol for 15 min and stained with 0.1% crystal violet for 20 min. Then, the colonies were photographed and counted.

### Flow cytometry

After treatment with 5-FU for 48 h, the stably transfected CRC cells were digested with DMSO-free trypsin (Invitrogen, Carlsbad, CA, USA) and washed two times with cold PBS. Then, flow cytometry (BD Biosciences, NJ, USA) was carried out after double staining with APC-annexin V and 7-amino-actinomycin D (7-AAD, BD Biosciences, NJ, USA) according to the manufacturer’s protocol.

### Western blotting assay and antibodies

Total cellular protein was collected in RIPA buffer (50 mM Tris pH 8.0, 150 mM NaCl, 1% NP-40, 0.5% sodium deoxycholate, 0.1% SDS) containing phenylmethylsulfonyl fluoride (PMSF), aprotinin, sodium orthovanadate and NaF. Equal amounts of proteins were subjected to electrophoresis on a SurePAGE gel (GenScript, Nanjing, China) and then transferred onto a polyvinylidene fluoride (PVDF) membrane. The membrane was then blocked in 5% skimmed milk for 1 h at room temperature and incubated with different diluted primary antibodies, including HMGA2 (1:1000 dilution, CST, MA, USA), PARP (1:1000 dilution, CST, MA, USA), cleaved PARP (1:1000 dilution, CST, MA, USA), caspase-3 (1:1000 dilution, CST, MA, USA), cleaved caspase-3 (1:1000 dilution, CST, MA, USA), caspase-7 (1:1000 dilution, CST, MA, USA), cleaved caspase-7 (1:1000 dilution, CST, MA, USA), P-ERK (1:2000 dilution, CST, MA, USA), ERK (1:1000 dilution, CST, MA, USA), tubulin (1:1000 dilution, CST, MA, USA), and GAPDH (1:1000 dilution, CST, MA, USA), at 4 °C overnight. Finally, an enhanced chemiluminescence (ECL) detection system (Merck, Darmstadt, Germany) and the Syngene GeneGenius gel imaging system (Syngene, Cambridge, UK) were used to visualize the protein bands after incubation with secondary antibody.

### Immunofluorescence staining

Transfected cells were seeded into 6-well plates placed with coverslips in the bottom and cultured overnight. Then, the culture medium was replaced with fresh culture medium containing 5-FU. After 48 h of incubation, the cells were fixed and permeabilized with a methanol and acetone mixture. After blocking with 10% goat serum, the cells were incubated with primary anti-cleaved caspase-3 (1:100 dilution, CST, MA, USA) or anti-Ki-67 (1:100 dilution, CST, MA, USA) at 4 °C overnight, followed by incubation with secondary Alexa Fluor 555 goat anti-mouse IgG (1:100 dilution, CST, MA, USA) or Alexa Fluor 647 goat anti-rabbit IgG (1:100 dilution, CST, MA, USA) for 1 h. Coverslips were mounted on slides using DAPI. The cells were visualized using a fluorescence microscope.

### Statistical analysis

All statistical analyses were performed with SPSS Version 25.0 software (IBM) and GraphPad Prism Version 7 software (GraphPad Software). Data are presented as the mean ± SD. The results were considered statistically significant at *p* < 0.05. The statistical differences within any two groups were used Student’s t test.

## Results

### CRART16 expression is upregulated in 5-FU-resistant CRC cells

**Fig. 1:**
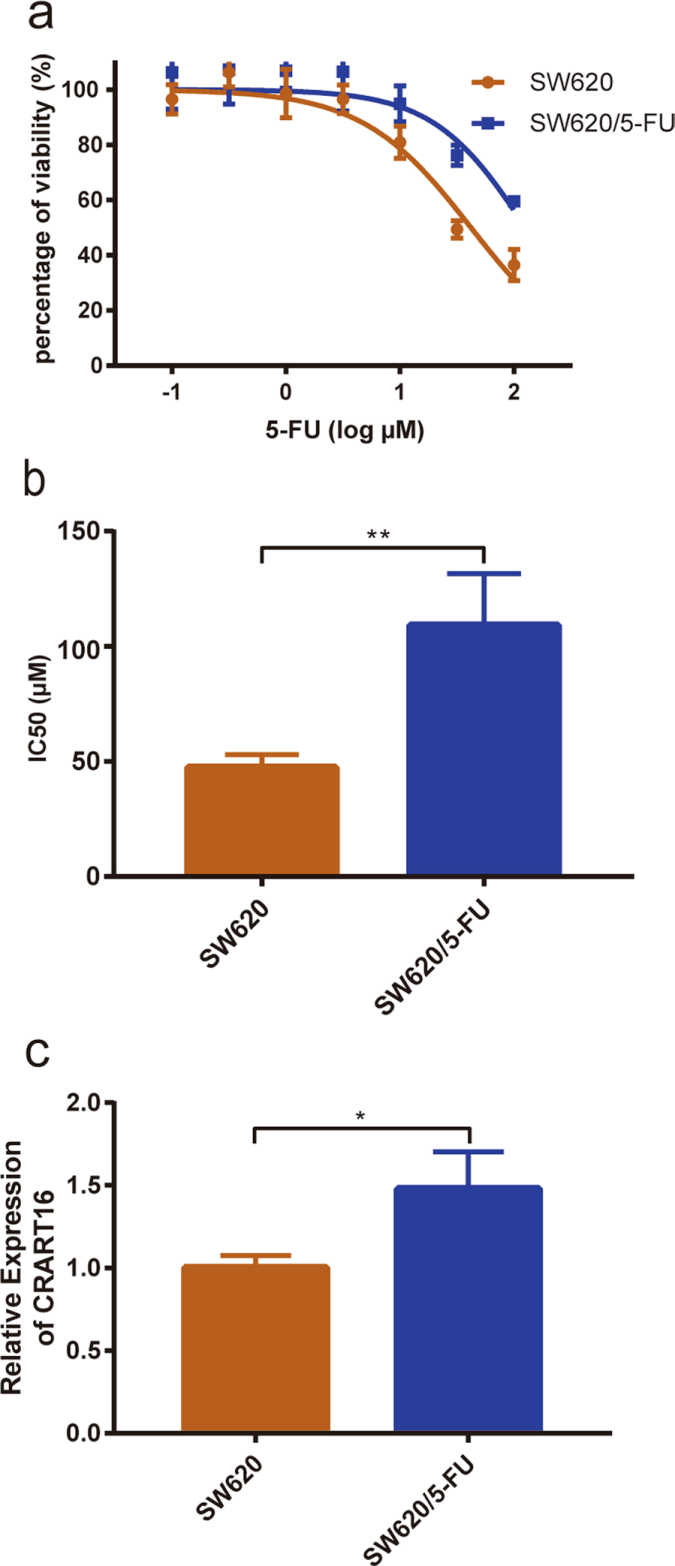
CRART16 is upregulated in SW620/5-FU cells. **a**, **b** The cytotoxic effect of 5-FU was evaluated by CCK-8 assay in SW620 and SW620/5-FU cells after exposure to graded concentrations of 5-FU for 48 h (**a**), and the IC50 values were calculated (**b**). **p < 0.01. **c** The expression levels of CRART16 in SW620 and SW620/5-FU cells were measured by RT-qPCR. GAPDH was used as an internal reference. Data are presented as the mean ± SD from three independent experiments. *p < 0.05

The expression levels of CRART16 in different CRC cell lines have been shown in our previous published study [17]. To detect whether CRART16 is involved in the acquired resistance of CRC cells to 5-FU, its expression level in a cell line with acquired 5-FU resistance was assessed. The 5-FU-resistant variant SW620/5-FU cell subline was generated by the stepwise screening of SW620 cells exposed continuously to increasing concentrations of 5-FU at a range of 0.1 to 10 µM for approximately 6 months. The CCK-8 assay was applied to confirm the sensitivity of SW620/5-FU and parent cells to 5-FU. Figure 1a shows that the cell viability rate of SW620/5-FU cells was significantly higher than that of SW620 cells when exposed to 5-FU. The half-maximal inhibitory concentration (IC50) value of 5-FU in SW620/5-FU cells was 109.20 ± 12.92 µM, while that in SW620 cells was 47.44 ± 3.17 µM (*p* < 0.01, Fig. 1b). Then, the expression levels of CRART16 were investigated by RT-qPCR in SW620/5-FU and parent cells. The results demonstrated that the expression of CRART16 was significantly upregulated in SW620/5-FU cells versus parental cells (Fig. 1c), suggesting that CRART16 may participate in 5-FU-acquired resistance in CRC cells.

### Overexpression of CRART16 is associated with 5-FU resistance in CRC cells

**Fig. 2:**
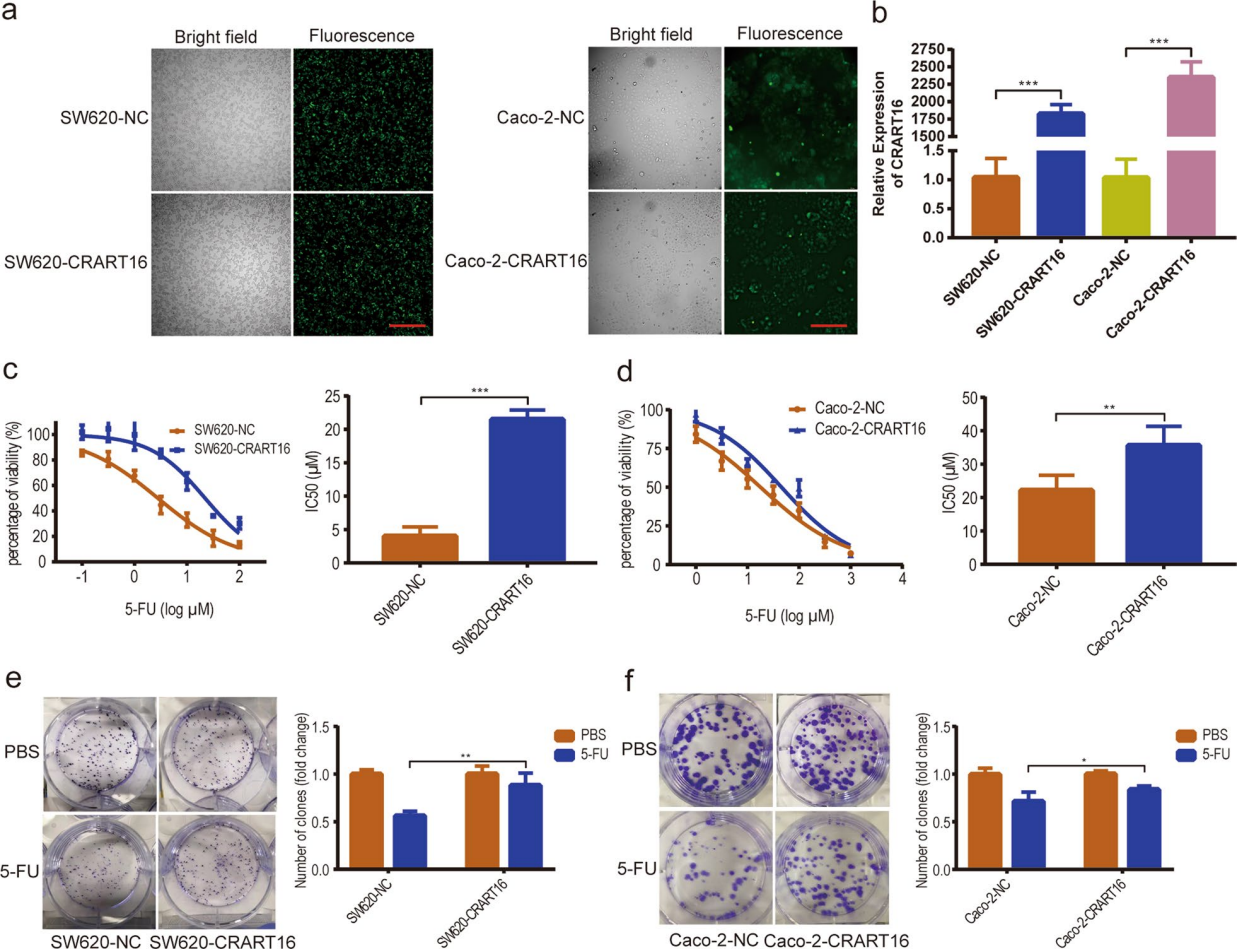
CRART16 confers 5-FU resistance in CRC cells. **a**, **b** Overexpression efficiency in CRART16 in SW620 and Caco-2 cells was validated by fluorescence microscopy (Scale bar: 100 μm) (**a**) and RT-qPCR (**b**). ***p < 0.001. **c**, **d** The sensitivity of SW620-NC and SW620-CRART16 cells (**c**) and Caco-2-NC and Caco-2-CRART16 cells (**d**) to 5-FU was assessed by CCK-8 assay after treatment with increasing concentrations of 5-FU for 48 h. The IC50 values of 5-FU were calculated. Data are presented as the mean ± SD from three independent experiments. ***p < 0.001, **p < 0.01. **e**, **f** Effects of CRART16 overexpression on colony formation of CRC cells treated with 5-FU. **p < 0.01, *p < 0.05

**Fig. 3:**
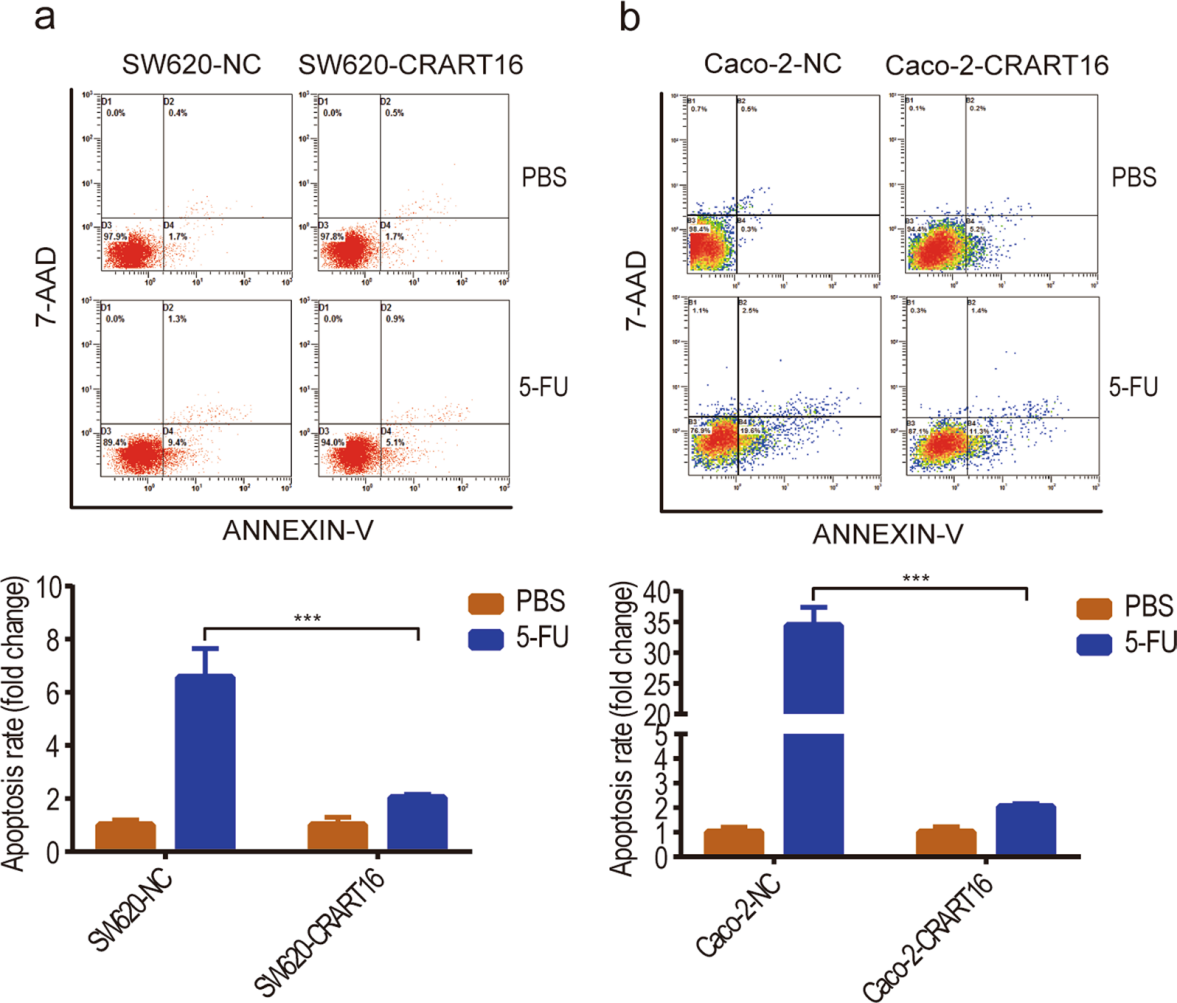
CRART16 confers 5-FU resistance in CRC cells. **a**, **b** The effects of CRART16 overexpression on cell apoptosis were detected by flow cytometry in CRC cells exposed to 5-FU. ***p < 0.001

**Fig. 4:**
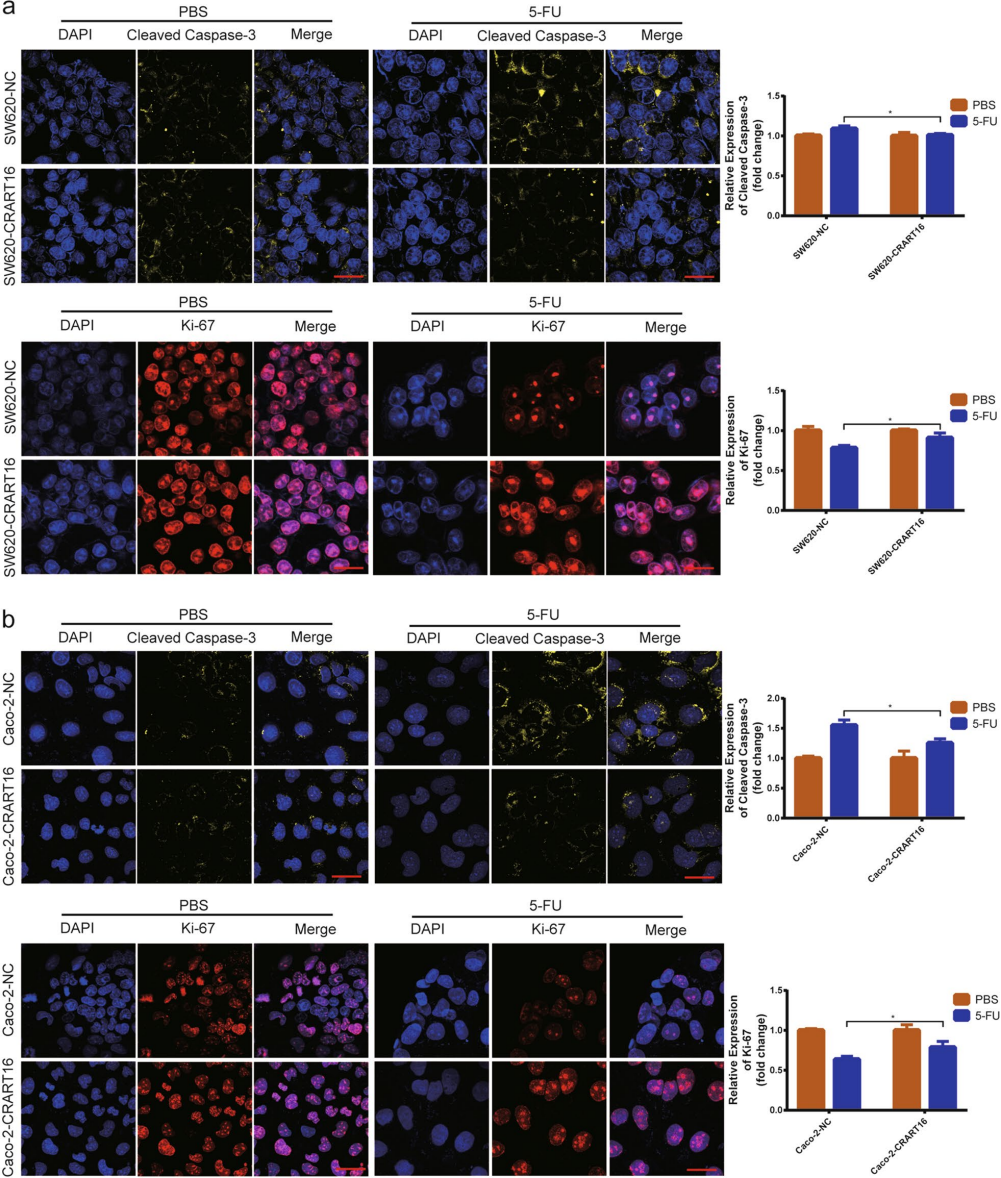
CRART16 confers 5-FU resistance in CRC cells. **a**, **b** The effects of CRART16 overexpression on the expression of cleaved caspase-3 and Ki-67 were assessed by immunofluorescence staining (Scale bar: 2 μm). *p < 0.05

To investigate the precise biological function of CRART16 in the 5-FU chemoresistance of CRC cells, we stably overexpressed CRART16 in SW620 and Caco-2 cells. Fluorescence microscopy and RT-qPCR were adopted to confirm the efficiency of transfection (Fig. 2a, b). After treatment with increasing concentrations of 5-FU for 48 h, cell viability was evaluated by CCK-8 assay (Fig. 2c, d). Compared with SW620-NC and Caco-2-NC cells, the IC50 values of 5-FU in CRART16-overexpressing cells were significantly increased by 434.58% and 60.96%, respectively (SW620-NC 4.02 ± 0.79 µM, SW620-CRART16 21.49 ± 0.82 µM, *P* < 0.001; Caco-2-NC 22.13 ± 2.04 µM, Caco-2-CRART16 35.62 ± 2.6 µM, *P* < 0.01). We performed a colony formation assay under 5-FU treatment and revealed that after CRART16 was upregulated, the number of cell clones was less suppressed by 5-FU (*P* < 0.01, *P* < 0.05, Fig. 2e, f). To further examine whether CRART16 had an effect on 5-FU-induced apoptosis, a flow cytometric assay was performed. The results showed that overexpression of CRART16 in SW620 and Caco-2 cells with 5-FU treatment had lower apoptotic rates than the negative control cells (Fig. 3a, b). Consistently, we observed that the apoptotic marker cleaved caspase-3 was downregulated and the proliferative marker Ki-67 was upregulated in CRART16-overexpressing cells versus negative control cells 48 h after 5-FU treatment (*P* < 0.05, Fig. 4a, b). Collectively, these data demonstrated that CRART16 could promote 5-FU resistance in CRC cells by attenuating apoptosis.

### CRART16 directly binds to miR-193b-5p as a competing endogenous RNA (ceRNA)

**Fig. 5:**
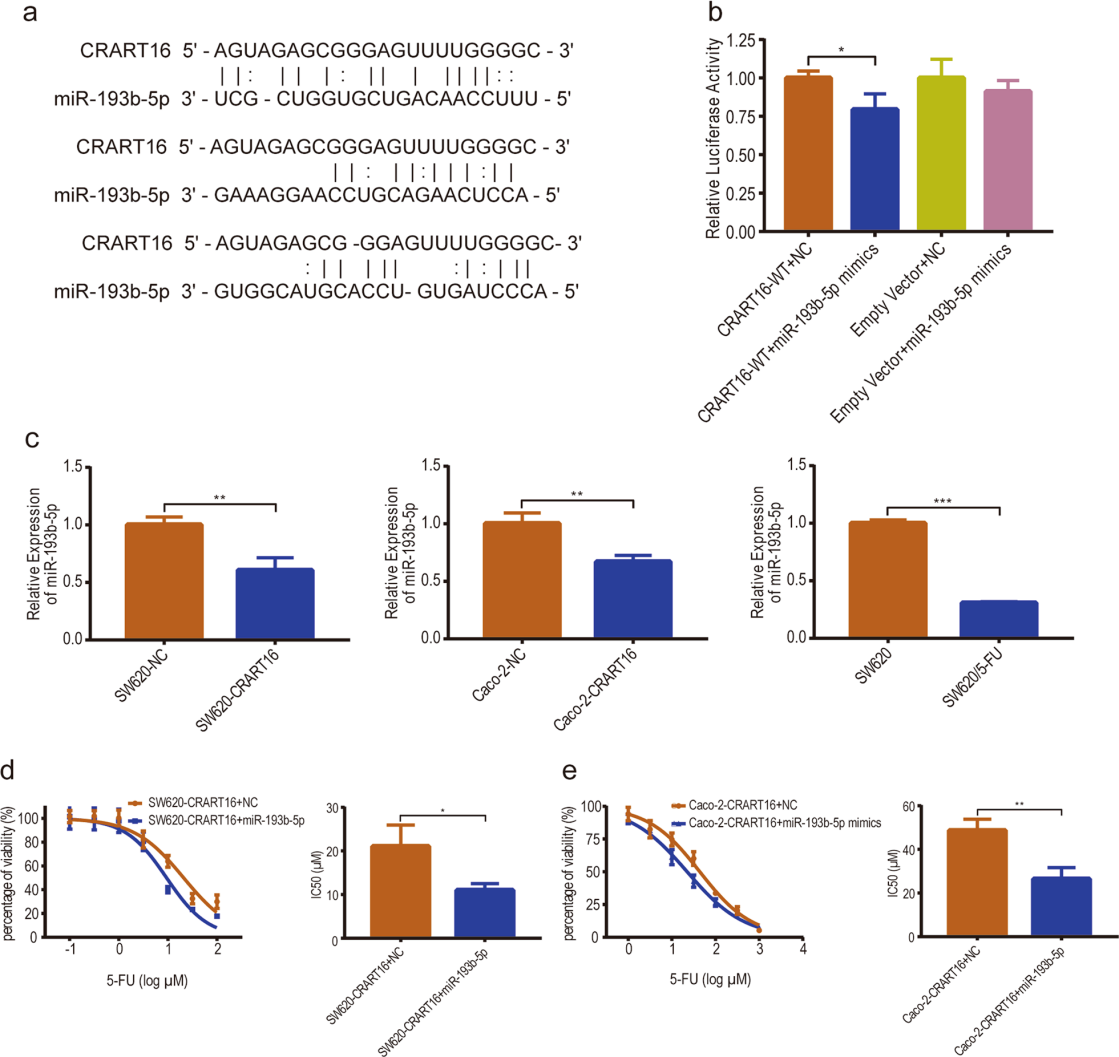
CRART16 binds to miR-193b-5p as a ceRNA. **a** Schematic illustration of miR-193b-5p binding sequences in CRART16. **b** The dual luciferase reporter assay was performed to measure the luciferase activity after co-transfection of 293 T cells with miR-193b-5p mimics or NC and pmiR-RB-Report™-CRART16-WT vector or empty vector. *p < 0.05. **c** The expression levels of miR-193b-5p in CRART16-overexpressing CRC cells, SW620/5-FU cells and their corresponding control cells were measured by RT-qPCR. U6 was used as an internal reference. Data are presented as the mean ± SD from three independent experiments. **p < 0.01, ***p < 0.001. **d**, **e** The viability of SW620-CRART16 and Caco-2-CRART16 cells transfected with miR-193b-5p mimics or NC was detected by CCK-8 assay after treatment with stepwise increasing concentrations of 5-FU for 48 h. The IC50 values of 5-FU were calculated. *p < 0.05, **p < 0.01

Our previously published study demonstrated that CRART16 could confer cetuximab resistance in CRC cells by functioning as a ceRNA [17]. We hypothesized that CRART16 could also promote 5-FU resistance in CRC cells by inhibiting the function of a certain miRNA. Based on the previous work of RNA-seq analysis and bioinformatics prediction, we found that CRART16 harbors several recognition sequences of miR-193b-5p (Fig. 5a). To confirm the potential relationship between CRART16 and miR-193b-5p, luciferase reporter vectors containing full-length CRART16 were constructed for the dual-luciferase reporter assay. We observed that compared with the miRNA negative control group, the miR-193b-5p mimics group showed dramatically reduced luciferase activity for the CRART16-WT reporter vectors but little change in that for the empty vectors (Fig. 5b). In addition, RT-qPCR was performed to assess the expression levels of miR-193b-5p, and the results showed that the expression levels of miR-193b-5p were significantly downregulated in CRART16-overexpressing cells and SW620/5-FU cells compared with the corresponding control cells (Fig. 5c). Taken together, these results revealed that the expression of miR-193b-5p was negatively regulated by CRART16 through direct binding. We further performed a rescue experiment to evaluate the function of miR-193b-5p in 5-FU resistance induced by CRART16 overexpression. The CCK-8 assay revealed that ectopic miR-193b-5p expression significantly reversed CRART16-induced 5-FU resistance in both SW620-CRART16 and Caco-2-CRART16 cells (SW620-CRART16 + NC 21.07 ± 2.79 µM, SW620-CRART16 + miR-193b-5p mimics 11.02 ± 0.86 µM, *p* < 0.05; Caco-2-CRART16 + NC 48.67 ± 2.98 µM, Caco-2-CRART16 + miR-193b-5p mimics 26.41 ± 3.0 µM, *p* < 0.01) (Fig. 5d, e). Collectively, these results demonstrated that CRART16 promoted 5-FU resistance by suppressing the expression of miR-193b-5p in CRC cells.

### CRART16 sponges miR-193b-5p to modulate HMGA2 expression

**Fig. 6:**
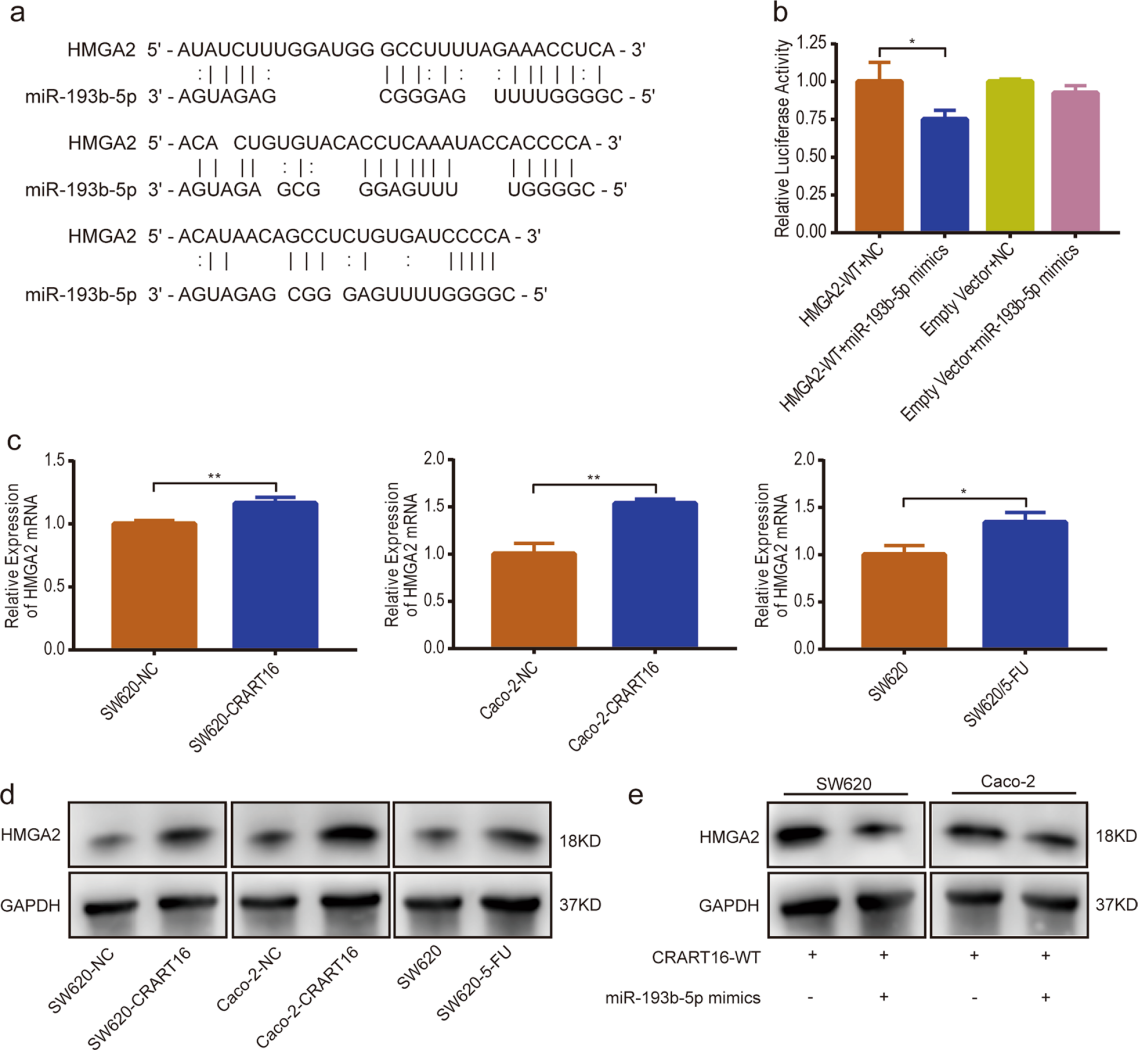
CRART16 sponges miR-193b-5p to modulate HMGA2 expression. **a** Schematic illustration of miR-193b-5p binding sequences in the HMGA2 3′ UTR. **b** The dual luciferase reporter assay was performed to measure the luciferase activity after co-transfection of miR-193b-5p mimics or NC with pmiR-RB-Report™-HMGA2 3′ UTR-WT vector or empty vector in 293 T cells. *p < 0.05. **c** The mRNA expression levels of HMGA2 in CRART16-overexpressing CRC cells, SW620/5-FU cells and their corresponding control cells were determined by RT-qPCR. *p < 0.05, **p < 0.01. **d** The protein expression levels of HMGA2 in CRART16-overexpressing CRC cells, SW620/5-FU cells and their corresponding control cells were assessed by western blotting. **e** The protein expression levels of HMGA2 in SW620-CRART16 and Caco-2-CRART16 cells transfected with miR-193b-5p mimics or NC were assessed by western blotting

Based on previous mRNA sequencing data and bioinformatics analysis results, we concentrated on HMGA2 as a potential target mRNA of miR-193b-5p. The binding sites between miR-193b-5p and the HMGA2 3′ untranslated region (UTR) predicted by TargetScan and RNAhybrid are displayed in Fig. 6a. The dual-luciferase reporter assay revealed that compared with the miRNA negative control group, overexpression of miR-193b-5p strikingly repressed the luciferase gene expression of HMGA2 3′ UTR reporter vectors while having little effect on empty vectors (Fig. 6b). The results indicated that HMGA2 was the target mRNA of miR-193b-5p. Subsequently, the expression levels of HMGA2 were assessed by RT-qPCR and western blotting. The results showed that HMGA2 expression was obviously upregulated in CRART16-overexpressing cells and SW620/5-FU cells (Fig. 6c, d). Furthermore, to detect whether CRART16 regulates HMGA2 expression via miR-193b-5p in CRC cells, a rescue experiment was performed and showed that introduction of miR-193b-5p mimics significantly attenuated HMGA2 protein levels in both SW620-CRART16 and Caco-2-CRART16 cells (Fig. 6e). Collectively, these results demonstrated that CRART16 sponges miR-193b-5p to upregulate HMGA2 expression in CRC cells.

### CRART16 may inhibit 5-FU-induced apoptosis through the MAPK signaling pathway

**Fig. 7:**
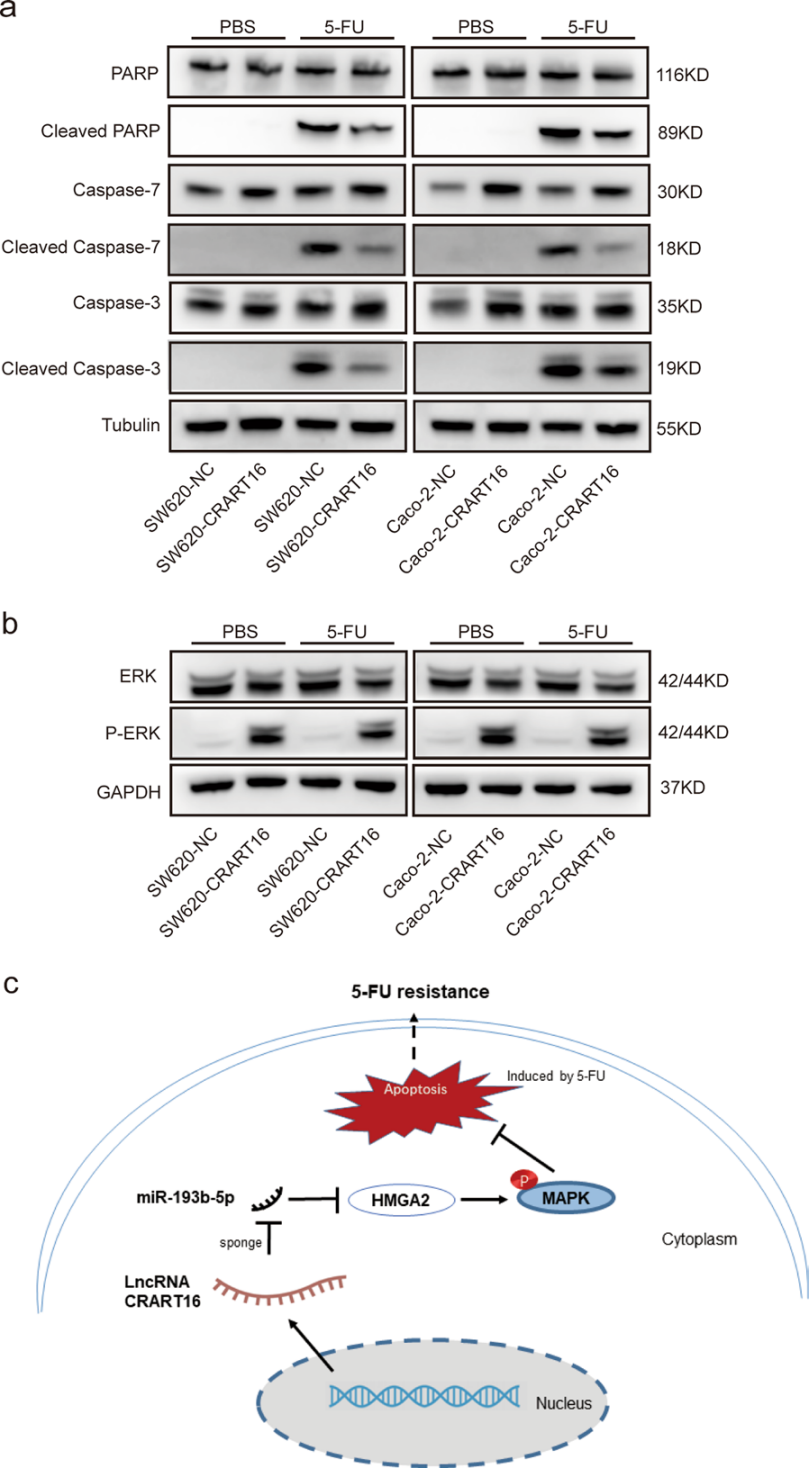
CRART16 activates the MAPK signaling pathway. **a** Cleaved and total PARP, caspase-3, caspase-7 levels were measured by western blotting. Tubulin was used as an internal reference. **b** ERK and p-ERK levels were measured by western blotting. GAPDH was used as an internal reference. **c** Schematic diagram of the mechanism of CRART16 in 5-FU resistance

In the present study, we demonstrated that CRART16 could promote 5-FU resistance in CRC cells by attenuating apoptosis. Western blotting further showed that CRART16 noticeably suppressed the levels of the apoptosis-related proteins cleaved PARP, cleaved caspase-3 and cleaved caspase-7 when exposed to 5-FU (Fig. 7a). In our previous study, based on RNA-seq, Gene Ontology (GO) enrichment and Kyoto Encyclopedia of Genes and Genomes (KEGG) pathway analysis, we found that the MAPK signaling pathway was obviously differentially enriched between Caco-2-CRART16 and Caco-2-NC cells [17]. In the present study, western blotting confirmed that the protein level of p-ERK was upregulated in both SW620-CRART16 and Caco-2-CRART16 cells (Fig. 7b), suggesting that the MAPK signaling pathway was activated by CRART16. Collectively, these results preliminarily showed that CRART16 attenuated 5-FU-induced apoptosis via the MAPK signaling pathway.

## Discussion

In this study, we identified that lncRNA CRART16 expression was strikingly upregulated in 5-FU-resistant CRC cells. Functionally, we demonstrated that CRART16 promoted 5-FU resistance in CRC cells by attenuating apoptosis. Further mechanistic study revealed that CRART16 exerts its functions by sponging miR-193b-5p in CRC cells. In addition, CRART16 modulates the expression of HMGA2 by inhibiting miR-193b-5p and activates the MAPK signaling pathway.

In recent years, several studies have indicated that lncRNAs play a vital role in 5-FU resistance. For instance, H19 is upregulated in recurrent CRC tissues and contributes to 5-FU resistance by sponging miR-194-5p and then promoting SIRT1-mediated autophagy in CRC [19]. Conversely, it is downregulated and abates 5-FU resistance through the miR-193a-3p/PSEN1 axis in hepatocellular carcinoma [20]. HOTAIR confers 5-FU resistance by sponging miR-218 and activating NF-kB/TS signaling [21]. Our previous work first found that CRART16 promotes cetuximab resistance by enhancing ERBB3 expression by binding to miR-371a-5p in CRC cells [17]. In the present study, we focused on the role of CRART16 in 5-FU resistance in CRC and found that it was markedly upregulated in 5-FU-resistant cells. Moreover, after overexpression of CRART16, 5-FU sensitivity was sufficiently attenuated, as indicated by significantly decreased apoptosis, suggesting that CRART16 contributes to 5-FU resistance in CRC cells by decreasing apoptosis.

Recent studies have shown that lncRNAs can suppress the expression of miRNAs by acting as ceRNA sponges [22]. MiRNAs, a class of ncRNAs with lengths of 21–25 nucleotides [23], decrease the expression and thereby prevent the translation of their downstream target mRNAs by binding to complementary sequences located in the 3′ UTRs of mRNAs [24]. Over the past two decades, the crucial roles of miRNAs in cancer have been discovered [25]. It has emerged that miRNAs can participate in 5-FU resistance [26]. MiR-135b and miR-182 contribute to 5-FU resistance in CRC by deregulating ST6GALNAC2 and further activating the PI3K/AKT pathway [27]. In vitro and in vivo, overexpression of miR-15b-5p increases 5-FU-induced apoptosis and enhances 5-FU sensitivity by negatively regulating its NF-κB1 and IKK-α targets [28]. Previously, CRART16 was shown to promote cetuximab resistance by sponging miR-371a-5p in CRC cells [17]. Due to the upregulation and chemoresistance role of CRART16, it is reasonable to assume that CRART16 promotes 5-FU resistance by sponging a certain tumor suppressive miRNA. Our results revealed that the transcript levels of CRART16 and miR-193b-5p were negatively correlated in CRC cells. The direct binding relationship between them was validated by a dual-luciferase reporter assay. Several studies have indicated that miR-193b-5p can function as a tumor suppressive miRNA in different malignancies, including gastric cancer [29], acute myeloid leukemia [30], and breast cancer [31]. Consistently, in our study, rescue experiments demonstrated that overexpression of miR-193b-5p significantly reversed CRART16-induced 5-FU resistance in CRC cells.

Our study established that CRART16 upregulates the expression of HMGA2 by sponging miR-193b-5p in CRC cells. HMGA2, a member of the high-mobility group A (HMGA) gene family, encodes a small non-histone chromatin-associated protein without intrinsic transcriptional activity that can modulate transcription by remodeling the chromatin architecture [32, 33]. The HMGA2 protein is normally expressed abundantly during embryogenesis, but its expression is hard to detect in terminally differentiated tissues [32, 34]. Numerous studies have revealed that HMGA2 is overexpressed in many cancer cells [35], and overexpression of HMGA2 is correlated with progression and a poor prognosis in various malignancies, including CRC [36–40]. Furthermore, several studies have demonstrated that HMGA2 participates in 5-FU resistance in some malignancies, such as CRC [39, 41, 42], breast cancer [43, 44], and liver cancer [45]. For example, Zheng et al. [41] revealed that HMGA2 could promote 5-FU resistance, which could be reversed by miR-9-5p, in CRC cells. Wu et al. [39, 42] indicated that HMGA2 expression was upregulated by lncRNA PCAT6-mediated sponging of miR-204, ultimately contributing to 5-FU resistance in CRC cells, which could be reversed by silencing HMGA2. Consistently, in our study, we detected that HMGA2 was upregulated in 5-FU-resistant CRC cells and was modulated by the direct binding of CRART16 to miR-193b-5p. Taken together, we conclude that CRART16 contributes to the 5-FU resistance of CRC cells by upregulating HMGA2 expression by suppressing miR-193b-5p.

In this study, we found that CRART16 could inhibit apoptosis induced by 5-FU in CRC cells. Consequently, we tried to reveal the mechanism by which CRART16 modulates apoptosis. Based on previous works involving RNA-seq and GO enrichment and KEGG pathway analysis, we found that mRNAs in the MAPK signaling pathway were remarkably enriched in Caco-2-CRART16 cells versus Caco-2-NC cells [17]. Several studies have demonstrated that the ERK/MAPK signaling pathway might be an essential pathway of apoptosis in different malignancies [46–48]. Consistent with a present study, we found that the protein level of p-ERK was upregulated in CRART16-overexpressing CRC cells. Collectively, these results suggest that CRART16 could activate the MAPK signaling pathway to inhibit 5-FU-induced apoptosis in CRC cells. Intriguingly, overexpression of HMGA2 was found to upregulate the expression level of p-ERK in prostate cancer cells [49]. In addition, the downregulation of p-ERK by shPP4R1 transfection was restored by HMGA2 overexpression in non-small-cell lung cancer (NSCLC), whereas HMGA2 silencing attenuated the expression level of p-ERK induced by PP4R1 overexpression [50]. Overall, we concluded that CRART16 inhibits apoptosis induced by 5-FU through the MAPK signaling pathway modulated by HMGA2 in CRC cells.

Although there are still some limitations in our study, it provides a direction for following research. Only two cell lines were used in this study, which may have a certain influence on the extrapolation of the experimental results. We will carry out validation experiments on primary cells and perform in vivo experiments in the follow-up works.

## Conclusions

In summary, CRART16 contributes to reducing the sensitivity of CRC cells to 5-FU by upregulating HMGA2 via suppression of miR-193b-5p, thereby activating the MAPK signaling pathway (Fig. 7c). Thus, CRART16 could be a promising therapeutic target for improving the effectiveness of 5-FU-based chemotherapy in CRC.

## Data Availability

The datasets generated during and/or analyzed during the current study are available from the corresponding author on reasonable request.

## Abbreviations

CCK-8: Cell counting kit-8
cDNA: Complementary DNA
ceRNA: Competing endogenous RNA
CRART16: Cetuximab resistance-associated RNA transcript 16
CRC: Colorectal cancer
DMEM: Dulbecco’s Modified Eagle Medium
ERBB3: V-Erb-B2 erythroblastic leukemia viral oncogene homologue 3
FBS: Fetal bovine serum
GAPDH: Glyceraldehyde 3-phosphate dehydrogenase
GO: Gene Ontology
HMGA2: High-mobility group AT-hook-2
KEGG: Kyoto Encyclopedia of Genes and Genomes
LncRNAs: Long noncoding RNAs
MAPK: Mitogen-activated protein kinases
mCRC: Metastatic colorectal cancer
NSCLC: Non-small-cell lung cancer
PMSF: Phenylmethylsulfonyl fluoride
PVDF: Polyvinylidene fluoride
RT-qPCR: Quantitative real-time PCR
TS: Thymidylate synthase
3′ UTR: 3′ Untranslated region
5-FU: 5-Fluorouracil
7-AAD: 7-Amino-actinomycin D
